# Band-gap engineering of zirconia by nitrogen doping in reactive HiPIMS: a step forward in developing innovative technologies for photocatalysts synthesis

**DOI:** 10.3389/fchem.2023.1239964

**Published:** 2023-08-10

**Authors:** Teodora Matei, Vasile Tiron, Roxana Jijie, Georgiana Bulai, Ioana-Laura Velicu, Daniel Cristea, Valentin Crăciun

**Affiliations:** ^1^ Faculty of Physics, Alexandru Ioan Cuza University of Iasi, Iasi, Romania; ^2^ Research Center on Advanced Materials and Technologies, Department of Exact and Natural Sciences, Institute of Interdisciplinary Research, Alexandru Ioan Cuza University of Iasi, Iasi, Romania; ^3^ Integrated Center of Environmental Science Studies in the North-Eastern Development Region (CERNESIM), Department of Exact and Natural Sciences, Institute of Interdisciplinary Research, Alexandru Ioan Cuza University of Iasi, Iasi, Romania; ^4^ Department of Materials Science, Faculty of Materials Science and Engineering, Transilvania University, Brasov, Romania; ^5^ National Institute for Laser, Plasma and Radiation Physics, Măgurele, Romania; ^6^ Extreme Light Infrastructure for Nuclear Physics, Măgurele, Romania

**Keywords:** reactive HiPIMS, band-gap engineering, photocatalytic activity, hysteresis effect, zirconium oxynitride thin film

## Abstract

In the global context of climate change and carbon neutrality, this work proposes a strategy to improve the light absorption of photocatalytic water-splitting materials into the visible spectrum by anion doping. In this framework, reactive high power impulse magnetron sputtering (HiPIMS) of a pure Zr target in Ar/N_2_/O_2_ gas mixture was used for the deposition of crystalline zirconium oxynitride (ZrO_2-x_N_x_) thin films with variable nitrogen doping concentration and energy band-gap. The nitrogen content into these films was controlled by the discharge pulsing frequency, which controls the target surface poisoning and peak discharge current. The role of the nitrogen doping on the optical, structural, and photocatalytic properties of ZrO_2-x_N_x_ films was investigated. UV-Vis-NIR spectroscopy was employed to investigate the optical properties and to assess the energy band-gap. Surface chemical analysis was performed using X-ray photoelectron spectroscopy, while structural analysis was carried out by X-ray diffraction. The increase in the pulse repetition frequency determined a build-up in the nitrogen content of the deposited ZrO_2-x_N_x_ thin films from ∼10 to ∼25 at.%. This leads to a narrowing of the optical band-gap energy from 3.43 to 2.20 eV and endorses efficient absorption of visible light. Owing to its narrow bandgap, ZrO_2-x_N_x_ thin films obtained by reactive HiPIMS can be used as visible light-driven photocatalyst. For the selected processing conditions (pulsing configuration and gas composition), it was found that reactive HiPIMS can suppress the hysteresis effect for a wide range of frequencies, leading to a stable deposition process with a smooth transition from compound to metal-sputtering mode.

## 1 Introduction

Solar energy, by utilizing various pathways for conversion, has the potential to play a vital role in the ambitious global pursuit of achieving net-zero greenhouse gas emissions by 2050. Despite claims from the harshest critics that the manufacturing of photovoltaic and photocatalytic integrated systems consumes significant amounts of energy and relies on the extraction of finite Earth minerals with severe environmental impacts, these technologies are continuously advancing. In fact, solar energy has already reached a point where it is cost-competitive with traditional methods of power generation. Furthermore, the implementation of effective circular economy strategies, along with the ongoing development of the mineral recycling industry, aims to minimize the negative consequences associated with mining. This makes solar energy an environmentally friendly technology, representing an extraordinary source of renewable and green energy. Photocatalytic technologies, such as solar fuels production, self-cleaning capabilities, and environmental remediation, have the potential to significantly contribute to the growth of sustainable energy markets.

In this context, zirconium oxide (ZrO_2_), commonly known as zirconia, stands out as an exceptional, unique, and highly versatile semiconductor. It finds widespread use due to its remarkable properties, including a high melting point, thermal stability, resistance to corrosion and hardness, chemical inertness, good biocompatibility, and optical transparency. The combination of these exceptional properties opens up a range of interesting and attractive applications. For instance, zirconia can be employed in indoor and outdoor architectural coatings, conventional and flexible optoelectronic devices (Venkataraj et al., 2002), shape memory alloys (Jette et al., 2021), and, of particular significance in the pursuit of decarbonization, as photocatalysts for hydrogen fuel production (Teeparthi et al., 2018; Besisa and Ewais, 2021).

However, when it comes to solar energy applications, zirconia photocatalysts face a challenge due to their wide band-gap [E_g_ ≃ 5.1 eV (Teeparthi et al., 2018)]. This characteristic leads to low efficiency in utilizing solar energy. In addition to charge separation and charge utilization efficiencies, the overall efficiency of a photocatalyst in converting solar energy depends directly on its ability to harness solar energy effectively. One approach to enhance this crucial aspect of a photocatalyst is by manipulating its band gap through band gap engineering, thereby extending its light absorption range from ultraviolet (UV) to visible light. One feasible method to achieve this is by introducing nitrogen as a dopant into the zirconium oxide lattice. This approach narrows the optical band gap, allowing for tailored optical performance and rendering the photocatalyst sensitive to visible light.

Strategies to produce Zr oxynitrides (ZrO_2-x_N_x_) involve two main approaches: controlled oxidation of zirconium nitride (Wiame et al., 1998) and nitridation of zirconia (Lerch, 1996). However, both of these methods typically require high processing temperatures, which can limit their application when dealing with heat-sensitive materials that need to be coated. In industrial-scale production, the synthesis of metal oxynitrides, including Zr oxynitrides, often relies on reactive magnetron sputtering. This method offers several advantages (Bräuer et al., 2010). Firstly, it allows for the deposition of high-density films and coatings with excellent thickness uniformity and strong adhesion to substrates. Additionally, reactive magnetron sputtering enables high deposition rates, providing efficient production capabilities. The process also offers good controllability, allowing for precise manipulation of the deposition parameters. Moreover, it demonstrates long-term stability, ensuring consistent and reliable outcomes. Furthermore, reactive magnetron sputtering is a relatively cost-effective deposition method, making it economically viable for large-scale production. Lastly, it offers the advantage of being able to uniformly coat large and heat-sensitive materials, expanding its potential applications.

Reactive magnetron sputtering is a process commonly used in thin film deposition. It involves sputtering a metallic target in a gas mixture of argon and a reactive gas. The compound coating is formed through the chemical reaction between the sputtered material and the reactive gas species at the substrate. The chemical composition of the resulting compound film can be controlled by adjusting the flux ratio of the sputtered material and the reactive gas. During reactive sputtering, particularly in the metal oxide mode, a phenomenon known as hysteresis effect can occur. This effect arises due to the formation of a compound film on the target surface and the inner walls of the deposition chamber. This effect can lead to process instability and significantly reduced sputtering efficiency (Greene, 2017). To ensure a stable and reproducible deposition process, it is necessary to suppress or eliminate this hysteresis effect. In addition to stabilizing the reactive deposition process, it is also important to control the transition between metal and compound-sputtering modes. This enables the growth of stoichiometric compound thin films at high deposition rates. When two different reactive gases are used, more complex processes come into play, including mixed target poisoning mechanisms (Van Bever et al., 2022), double hysteresis (Depla et al., 2006), and “process trapping” effects (Báranková et al., 1995). In such cases, experimental findings have shown that the transition zone and the width of the hysteresis loop depend strongly on the mass flow rates of oxygen and nitrogen (Martin and Rousselot, 1999). However, Severin et al. (2006) demonstrated that by using two reactive gases, such as oxygen and nitrogen, instead of just one (e.g., oxygen), it is possible to stabilize the transition zone.

The operation of magnetron sputtering in a high-power pulsed regime offers several advantages, including the ability to achieve transient regimes where physical and chemical processes can rapidly evolve under optimal conditions. This ensures excellent process stability and reproducibility. High-power impulse magnetron sputtering (HiPIMS) is a variant of this technique, characterized by the application of unipolar power pulses with high magnitude, low frequency, and low duty cycle to the conventional sputtering magnetron cathode (Kouznetsov et al., 1999). These pulses result in a high instantaneous plasma density. The elevated plasma density in HiPIMS facilitates the generation of highly ionized fluxes consisting of both metal and gas species, whose energy and direction can be controlled during the film deposition process using electric and/or magnetic fields (Tiron et al., 2012). The energetic ion bombardment occurring during film deposition enhances the synthesis of materials with superior properties compared to those obtained through conventional magnetron sputtering (Sarakinos et al., 2010). When compared to reactive direct current magnetron sputtering (dcMS), reactive HiPIMS, if configured properly, allows for the suppression or elimination of the undesired hysteresis effect without the need for feedback control systems (Sarakinos et al., 2008; Aiempanakit et al., 2011). Wallin et al. propose that the elimination or suppression of the hysteresis effect during reactive HiPIMS could be attributed to factors such as high erosion rate during the pulse, limited target oxidation between pulses, and gas rarefaction effects in front of the target (Wallin and Helmersson, 2008).

In terms of process stability, one of the challenges encountered in the reactive HiPIMS process is the occurrence of arcing. However, there are strategies to suppress both arcing and hysteresis effects in reactive HiPIMS, and one such strategy is the use of short pulses. By employing short pulse durations, there is less time for charge accumulation, which effectively suppresses the development of arcing on the target surface (Tiron et al., 2020). Additionally, when maintaining a constant average power, shorter pulse durations result in higher pulsing frequencies. This, in turn, reduces target poisoning between pulses (Tiron and Sirghi, 2015), thereby suppressing the hysteresis effect. Furthermore, when aiming to synthesize highly efficient photocatalysts for sustainable development goals, it is crucial to consider environmentally friendly and versatile manufacturing technologies. HiPIMS is well-regarded in the coating industry as an excellent green and eco-friendly processing technology. It not only offers an attractive combination of properties and performances but also aligns with the goal of utilizing environmentally friendly manufacturing techniques.

The approach adopted in this study to address the hysteresis effect and control the nitrogen incorporation in zirconia films involves the use of reactive HiPIMS conditions, an optimal gas mixture of Ar-O_2_-N_2_, and a suitable pulsing configuration. This strategy has been previously employed to finely adjust the nitrogen content and optical band-gap in other transition metal oxynitrides, such as ZnO_x_N_y_ (Tiron and Sirghi, 2015; Tiron et al., 2017) and TiO_x_N_y_ (Demeter et al., 2017).

A crucial factor in controlling the nitrogen content is the low oxygen content in the gas mixture. By introducing a low amount of oxygen into the discharge, the incorporation of oxygen atoms into the coating is limited, allowing the un-oxidized metal atoms to bond with the less reactive nitrogen atoms.

Additionally, the use of short HiPIMS pulses plays a significant role in suppressing both the hysteresis effect and arcing, leading to enhanced process stability. These benefits are achieved without the need for specific approaches (Vlček et al., 2013) or feedback control systems (Sproul et al., 2005).

The photocatalytic activity of the ZrO_2-x_N_x_ thin films was evaluated through the photodegradation tests. The results provide insight into the films’ ability to facilitate the degradation of the MB dye under visible-light exposure, demonstrating their potential for practical applications in photocatalysis.

## 2 Experimental details

The experiments were conducted in a high vacuum stainless steel chamber using specific equipment and parameters. A Zr sputtering target with a diameter of 50 mm and a thickness of 3 mm, possessing a purity of 99.95%, was utilized. The target was mounted on a 2" magnetron cathode configured with an unbalanced type II magnetic field, according to Window and Savvides classification (Window and Savvides, 1986).

A gas mixture comprising of high-purity argon (99.999%, flow rate of 50 sccm), nitrogen (99.999%, flow rate of 1 sccm), and oxygen (99.999%, flow rate of 0.1 sccm) was employed as the sputtering gas. The total pressure within the chamber was set to 0.8 Pa. To generate the required electrical pulses, a pulsing unit (home-built high-power pulse generator) was utilized, receiving a constant voltage from a DC power supply. The applied pulses were unipolar HiPIMS pulses with a consistent duration of 10 μs and a constant amplitude of −700 V. Various repetition frequencies were employed, ranging from 300 to 1500 Hz.

ZrO_2-x_N_x_ thin films were deposited onto Si (100) and quartz substrates placed 7 cm away from the target. For comparison, a reference ZrO_2_ film was synthesized using a pulse repetition frequency of 800 Hz and flow rates of 50 sccm for argon, 1 sccm for oxygen, and 0 sccm for nitrogen. The deposition process did not involve any substrate bias or heating. The deposition time was set to 30 min, and the deposition rate was measured using a quartz crystal microbalance rate monitor (Q-pod, Inficon), which was positioned near the port-substrate location. Before the thin film deposition at a specific repetition frequency, a 5-min target pre-sputtering process was conducted. This step aimed to eliminate any contaminants present on the target surface and to establish stable operational conditions for the deposition process.

During the experiments, the discharge voltage and current were monitored using a digital oscilloscope (Le Croy WaveSurfer 434) equipped with a high-voltage probe (TesTec, 1:100) and a current probe (Pearson, 1 V/1A). These probes were connected to the magnetron feedthrough to capture the time evolution of the discharge parameters.

To investigate process stability and understand the relationship between the film’s stoichiometry and target coverage, time-resolved optical emission spectroscopy (TR-OES) measurements were performed (Tiron et al., 2020). The optical radiation emitted by the entire plasma was concentrated onto the aperture of the monochromator using a collimating lens. Subsequently, an optical fibre was installed outside the vacuum chamber, directed towards the target surface, to transmit the focused radiation to the monochromator for further analysis. TR-OES allows for the analysis of the optical emission spectra over time, providing valuable insights into the stability of the deposition process and its impact on the resulting film’s composition.

The optical transmittance of the deposited films on quartz substrates was measured across a wide wavelength range of 190–1100 nm. This measurement was carried out using a UV-Vis-NIR spectrophotometer (Evolution 300 from Thermo Scientific). The transmittance measurements enabled the evaluation of the films’ optical behaviour, and the energy band-gap (E_g_) was determined through Tauc plot analysis. This analysis helps to understand the film’s optical properties, particularly the energy required for electronic transitions.

The chemical composition of the deposited films was analysed using X-ray Photoelectron Spectroscopy (XPS) with an ESCALB Xi + XPS system. Monochromatic AlK_α_ radiation was used as the X-ray source. Initially, survey scans were performed, consisting of 5 scans with a pass energy of 50.0 eV and a step size of 0.5 eV. This was followed by high-resolution scans of the core level regions of Zr, N, C, and O, with 5 scans at a pass energy of 20.0 eV and a step size of 0.1 eV.

To remove surface contaminants, a 5-min 500 eV Ar ion sputtering step was conducted, and the XPS acquisition was repeated. The binding energies of the XPS spectra were referenced to the adventitious C1s position at 284.8 eV, providing a standard reference point for accurate analysis.

X-ray diffraction (XRD) measurements were employed to investigate the structural properties and phase analysis of the samples. The XRD patterns were recorded in the 20°-80° 2θ range, with an angle step of 0.02° and a scan speed of 1°/min. A Shimadzu LabX XRD-6000 diffractometer equipped with a CuK_α_ radiation source (λ = 1.54059 Å) was used in Bragg-Brentano configuration.

To evaluate the photocatalytic efficiency of the synthesized ZrO_2-_x films, the degradation of MB under visible light irradiation was performed. The ZrO_x_N_y_ films, deposited on 1 cm × 1 cm quartz substrates, were placed in a PS well with a diameter of 15.6 mm. The PS well contained 3 mL of MB solution with a concentration of 12 μg/mL, obtained from Sigma Aldrich, Darmstadt, Germany.

The experimental setup involved illuminating the ZrO_2-x_N_x_ films with a visible lamp emitting light with a wavelength greater than 400 nm. The incident light flux was maintained at 25 mW/cm^2^ (Demeter et al., 2017). The photocatalytic reaction took place in ambient air. Before the irradiation process, the MB solution with the ZrO_2-x_N_x_ films was kept in the dark for 30 min to establish an absorption-desorption equilibrium.

The change in MB concentration over time during the irradiation process was monitored using a Specord 210 Plus UV-Vis spectrophotometer from Analytik Jena, Germany. The concentration values were determined based on a constructed calibration graph (y = 0.083 + 0.091x) specific to the MB dye. The degradation efficiency of the photocatalytic process was calculated using the following equation:

$$Degradation\left(\%\right)=\frac{C_{0}-C}{C_{0}}\times 100$$ (1)

where $C_{0}$ is the initial concentration of dye after 30 min stored in dark conditions, while C is the dye concentration after a certain irradiation time.

## 3 Results and discussion

### 3.1 Process stabilization and stoichiometry control

#### 3.1.1 Deposition rate

Understanding the behaviour of the reactive HiPIMS process is crucial for controlling process stability, deposition rate, and the properties of the deposited films, especially their chemical composition. In this study, the relationship between target coverage and process stability, specifically the hysteresis effect, was investigated by studying the influence of pulse repetition frequency on the deposition rate. The pulse frequency was varied between 300 and 1500 Hz to observe the transition from compound (oxide) sputtering mode to metal-sputtering mode. It is important to note that all the ZrO_2-x_N_x_ films were deposited at repetition frequencies within the transition zone of 800–1200 Hz.

**FIGURE 1:**
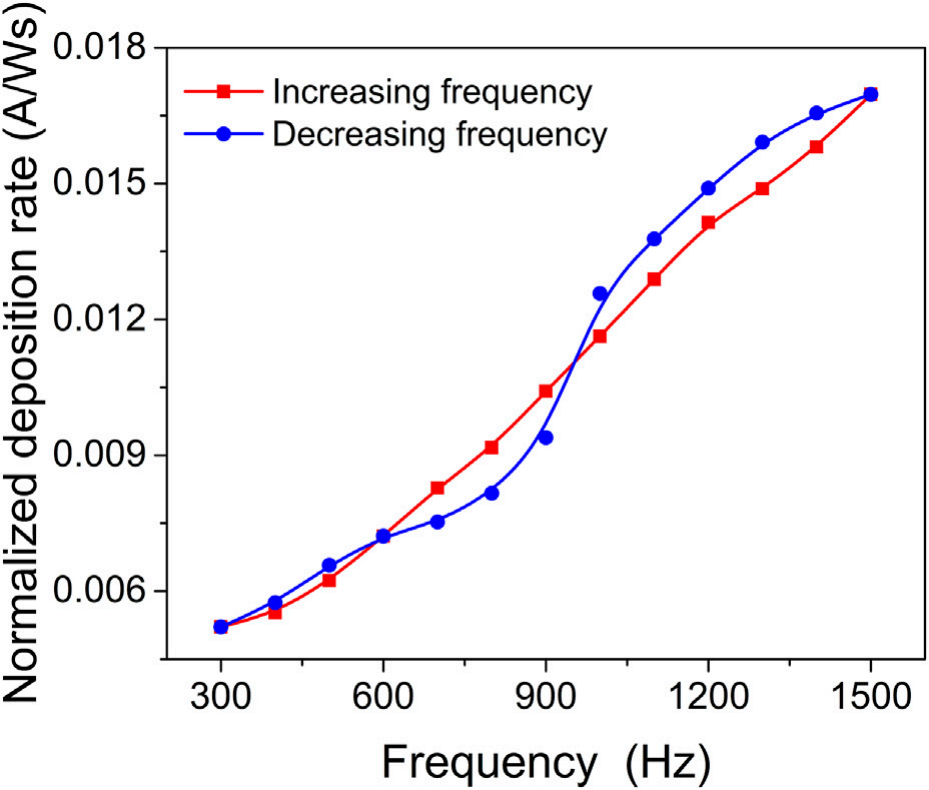
Deposition rate normalized to the average power applied to the target as a function of pulsing frequency.

Figure 1 depicts the influence of pulse repetition frequency on the deposition rate. As the average power of the HiPIMS discharge changed with the pulsing frequency (while pulse duration and amplitude were kept constant), the deposition rate was normalized to the average power. By varying the pulsing repetition frequency, a suppression of the hysteresis effect was observed, along with a smooth transition from the poisoned to metallic sputtering target mode.

These findings indicate that adjusting the pulse repetition frequency in the reactive HiPIMS process can effectively control the hysteresis effect and achieve a stable deposition process. The transition from compound sputtering mode to metallic sputtering mode can be achieved by selecting an appropriate pulsing repetition frequency. This knowledge is valuable for optimizing the deposition process and obtaining films with desired properties and chemical composition.

The suppression of hysteresis in the reactive HiPIMS discharge can be attributed to several factors. Firstly, the high erosion rate during the pulse and limited target poisoning between pulses contribute to reduced target poisoning (Sarakinos et al., 2008; Wallin and Helmersson, 2008). This means that the target surface is effectively cleaned during the pulses, and there is less build-up of deposited material between pulses. Additionally, the gas rarefaction effect, which occurs when the time between pulses is short enough to prevent gas refill in front of the target, can also contribute to hysteresis suppression (Kubart et al., 2011). This effect becomes more pronounced with increasing peak current.

In this study, as the pulse frequency increases, the power-normalized deposition rate gradually increases due to the increase in the target’s effective sputtering yield. It is important to note that a small amount of oxygen was added to the sputtering gas mixture, resulting in a coexistence of a nitride and an oxide compound system at the target surface. The sputtering yield of the nitride is higher than that of the corresponding oxide due to the lower binding energy of the nitride. As the pulsing frequency increases, the oxide compound is gradually removed from the target surface, shifting the surface composition from predominantly metal oxide to predominantly metal nitride, and finally to predominantly metal. This transition leads to an increase in the effective sputtering yield.

The high erosion rate during the pulses effectively cleans the target surface, while the reduced plasma off-time and limited plasma activation during the pulse off-time contribute to decreased target poisoning (Sarakinos et al., 2008). The synergistic effect between the increase in effective sputtering yield and the decrease in ion peak current (reduced ion return effect) results in a maximum deposition rate observed at high pulsing repetition frequencies.

Overall, by increasing the pulsing frequency in the reactive HiPIMS process, the hysteresis effect is suppressed through enhanced target surface cleaning, reduced target poisoning, and a favourable shift in the target surface composition towards metal nitride. These factors contribute to an increased deposition rate and improved process stability.

#### 3.1.2 Plasma emission

**FIGURE 2:**
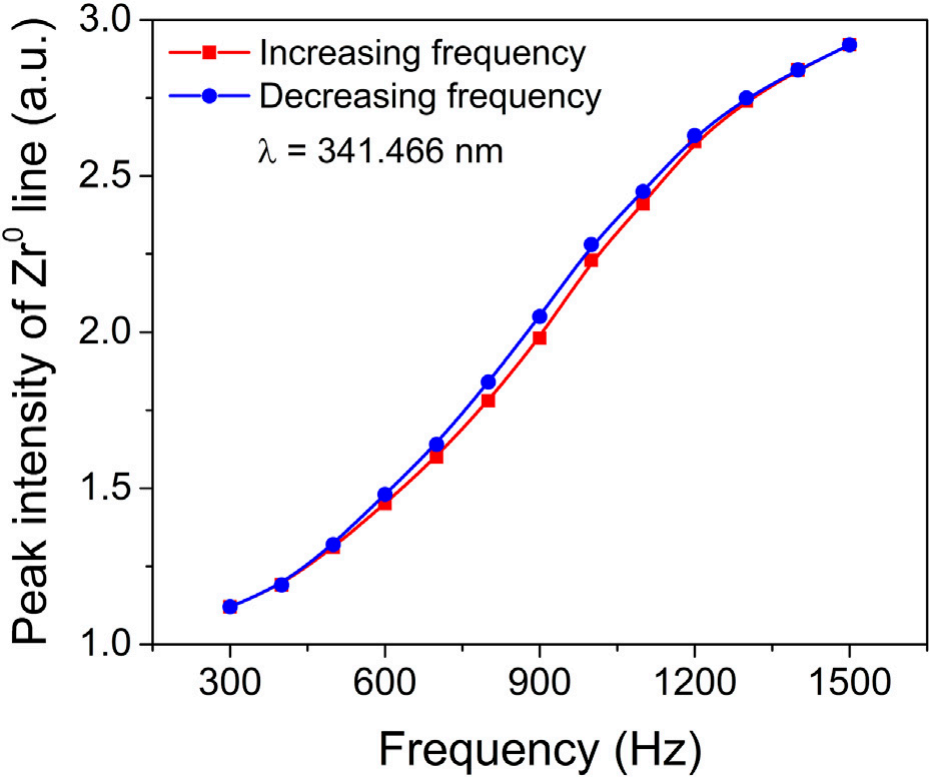
Peak intensity of Zr^0^ (341.466 nm) optical emission spectral line as a function of pulsing frequency.

In order to gain a more comprehensive understanding of the hysteresis behaviour and process stability, a detailed investigation was carried out using time-resolved optical emission spectroscopy (TR-OES). The main objective was to explore the relationship between the deposition rate and the coverage of the target material. TR-OES proved to be a highly valuable technique in this study, as it provided crucial insights into the coverage of the target compound, which is directly related to the abundance of excited metallic species present in the plasma. To establish a correlation between the deposition rate and target coverage, the intensity of a specific Zr optical emission line at 341.466 nm was carefully recorded at various pulsing frequencies. This particular Zr spectral line is known to exhibit the highest intensity in plasma optical emission spectroscopy (Figure S1). By examining Figure 2, which illustrates the influence of pulsing frequency on the peak intensity of the Zr optical emission spectral line, we can observe the significant impact that varying pulsing frequencies have on the observed intensity levels. This comprehensive investigation utilizing TR-OES and the analysis of the Zr optical emission line intensity provides valuable insights into the intricate interplay between the deposition rate, target coverage, and the pulsing frequency. Such understanding is crucial for optimizing process stability and further enhancing our knowledge of hysteresis behaviour in the studied system.

**FIGURE 3:**
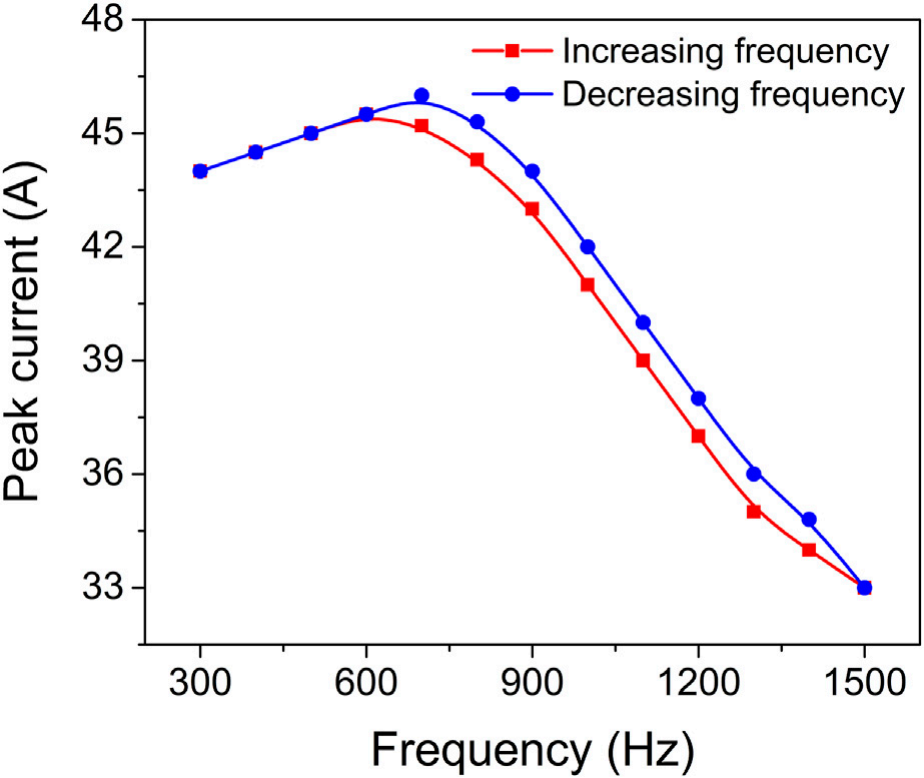
Peak current as a function of pulsing frequency.

Figure 3 depicts the gradual decrease in peak current within the frequency range of 700–1500 Hz. Interestingly, despite this trend, the peak intensity of the Zr0 optical emission spectral line exhibits a gradual increase as the pulse frequency rises. This implies that the intensity of the Zr^0^ optical emission line during the pulse, and consequently the presence of Zr atoms in the gas phase, further intensify with higher pulse repetition frequencies. The observed increase in pulse frequency results in the gradual removal of the oxide compound from the target surface. Therefore, the surface transitions from being predominantly covered by metal oxide to being predominantly covered by metal nitride, and eventually to being predominantly covered by metal. The smooth transition depicted in Figure 2 demonstrates that this shift from oxide sputtering to metal sputtering occurs without any sudden jumps or steep variations, indicating a remarkably stable process. This analysis highlights the dynamic relationship between pulse frequency, target surface composition, and the resulting emission behaviour. The findings provide valuable insights into the hysteresis behaviour and process stability, shedding light on the intricate mechanisms governing the transition from oxide to metal sputtering modes.

#### 3.1.3 Discharge peak current

To further explore the impact of deposition conditions on target coverage and process stability, the target peak current was measured as a function of pulse repetition frequency. Time evolution of the target voltage and discharge current during HiPIMS of Zr target in argon, nitrogen and oxygen gas mixture at pressure 0.8 Pa and repetition frequency 1 kHz are depicted in Supplementary Figure S2. Figure 3 illustrates the results of this investigation. By keeping the pulse duration constant at a specific value, it was observed that varying the pulse frequency led to a corresponding change in the peak current. In particular, increasing the pulsing frequency from 700 to 1500 Hz resulted in a linear increase in the average power, ranging from 80 to 140 W, while simultaneously causing a linear decrease in the peak current, from 46 to 33 A. This trend indicates a smooth transition from a predominantly oxidized target to a predominantly nitrided one. The narrow hysteresis loop, spanning only 1 A, along with the monotonic and linear relationship between peak current and pulsing frequency, point towards excellent process stability. Consequently, within this specific frequency range, the reactive HiPIMS technology offers a remarkable capability to control the chemical composition of ZrO_2-x_N_x_ thin films through the modulation of peak current. This insight provides valuable opportunities to precisely tailor the properties of the thin films based on the desired chemical composition, enabling enhanced control over the overall performance and functionality of the materials.

The difference in discharge current behaviour between the compound and metal-sputtering modes can be attributed to the varying target surface coverage by oxide compounds, which has a significant impact on the secondary electron emission yield (Hippler et al., 2023).

In this work, we have observed a somewhat surprising phenomenon where the peak current during predominantly oxide mode HiPIMS of a Zr target is higher compared to the peak current during predominantly nitride mode HiPIMS. This finding contrasts with the results reported by Depla et al. (2009), who found that during reactive DCMS of a Zr target, the secondary electron emission yield (SEEY) of Zr oxide is lower than that of Zr nitride and much lower than that of Zr metal. As a result, for the same applied voltage, the discharge current should be lower during the oxide mode DCMS of a Zr target.

Interestingly, our results are more consistent with the findings reported by Aiempanakit et al. (2013) for the oxide mode HiPIMS of a Ti target. They discovered a significant difference in the discharge behavior between DCMS and HiPIMS. In oxide mode HiPIMS of a Ti target, they observed a peak current that is much higher (about two times) compared to the metal mode HiPIMS of pure Ti target.

To explain the difference in discharge behavior between DCMS and HiPIMS, Aiempanakit et al. (2013) conducted ion mass spectrometry measurements. They found that during reactive DCMS, the discharge is primarily governed by Ar^+^ induced SEEY of the target surface. However, during reactive HiPIMS, the discharge is mainly governed by O^+^ induced SEEY of the target surface. The increased fraction of O^+^ ions in the total ionic current to the target, coupled with preferential sputtering of oxygen atoms and O^+^ ion back-attraction, results in a large ionic current and an increased effective SEEY. Additionally, the self-sputtering by O^+^ ions of an oxidized target leads to an increased release of secondary electrons due to the high ionization potential of 13.62 eV for O^+^.

These findings provide valuable insights into the complex discharge behavior and secondary electron emission processes occurring during oxide mode HiPIMS and help to understand the observed peak current differences between oxide and nitride modes of HiPIMS for different target materials.

Based on the reported findings by Aiempanakit et al. (2013) for the oxide sputtering mode of a Ti target, it is evident that the behaviour of reactive HiPIMS of Zr target can differ from conventional dcMS. In the case of reactive HiPIMS, the higher discharge peak current observed in the oxide-sputtering mode compared to the metal/nitride-sputtering regime can be attributed to the contribution of two factors: the O^1+^ ion recycling process and the higher O^1+^ induced secondary electron emission yield (SEEY) of the oxidized Zr target surface.

During the predominantly oxide sputtering mode, there is a steep increase in the oxygen ion concentration, likely caused by the reduced sputtering yield of the oxidized metal and the limited amount of metal available (Aiempanakit et al., 2013). This is accompanied by a steep increase in the discharge current. The preferential sputtering of oxygen from the target surface, driven by the high oxygen partial sputtering yields, leads to the replacement of sputtered oxygen atoms by back-attracted O^1+^ ions. This dynamic process affects the target cleaning and contributes to the O^1+^ induced SEEY of the oxidized target, which is higher compared to the metal mode.

Since the Zr target exhibits similar behaviour to the Ti target in terms of secondary electron emission yield (SEEY), it can be expected that the same discharge current behaviour occurs during the predominantly oxide mode HiPIMS of the Zr target. As the pulsing frequency increases, the higher amount of Zr atoms available leads to more oxygen consumption, resulting in less target oxidation and a lower O^1+^ induced SEEY. Consequently, the target peak current decreases. Therefore, the higher discharge peak current observed in the oxide-sputtering mode compared to the metal/nitride-sputtering mode in reactive HiPIMS is likely influenced by the interplay of the O^1+^ ion recycling process, the higher O^1+^ induced SEEY of the oxidized target surface, and the dynamics of target oxidation and consumption. However, as values O^1+^ and Ar^+^ induced SEEY of the oxidized Zr target surface are not known exactly, the results provide qualitative rather than quantitative information.

### 3.2 Thin films properties

#### 3.2.1 Optical properties

**FIGURE 4:**
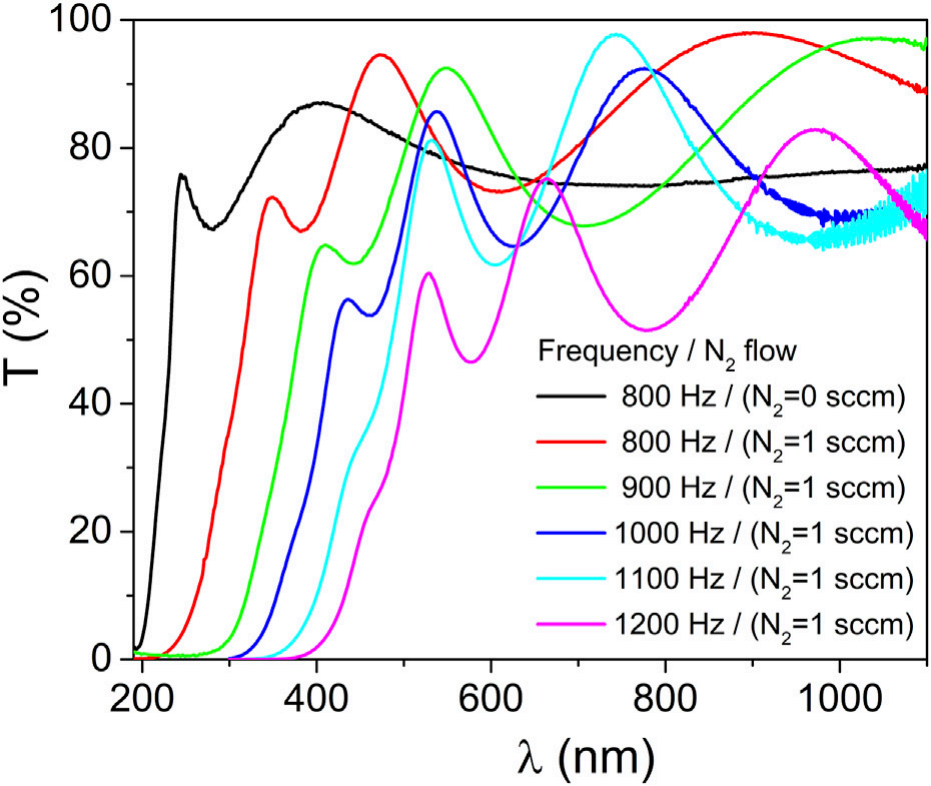
Optical transmittance of the ZrO_2_ and ZrO_2-x_N_x_ coatings prepared at different pulsing repetition frequencies.

The optical properties of the ZrO_2_ and ZrO_2-x_N_x_ thin films were characterized using transmittance measurements in the wavelength range of 190–1100 nm. Figure 4 presents the optical transmittance spectra of the thin films deposited at various pulsing repetition frequency values, ranging from 800 to 1200 Hz. The reference spectrum corresponds to the optical transmittance of a zirconia (ZrO_2_) thin film deposited at 800 Hz.

As depicted in Figure 4, a clear observation can be made regarding the optical properties of the ZrO_2_ and ZrO_2-x_N_x_ films. It is evident that the optical absorption edge gradually shifts towards longer wavelengths as the HiPIMS pulse repetition frequency increases. This shift indicates a decrease in the band-gap energy of the ZrO_2-x_N_x_ films with higher repetition frequencies. The change in band-gap energy suggests a modification in the electronic structure of the films, potentially leading to enhanced light absorption in the visible range.

In addition to the shifting absorption edge, another notable feature in Figure 4 is the variation in the interference fringes. With increasing pulse repetition frequency, the separation between consecutive maxima of the interference fringes decreases. This observation implies that the thickness of the ZrO_2-x_N_x_ thin films increases as the repetition frequency is raised. The controlled tuning of film thickness through pulsing parameters is of significant interest in tailoring the optical and functional properties of thin films for various applications.

**FIGURE 5:**
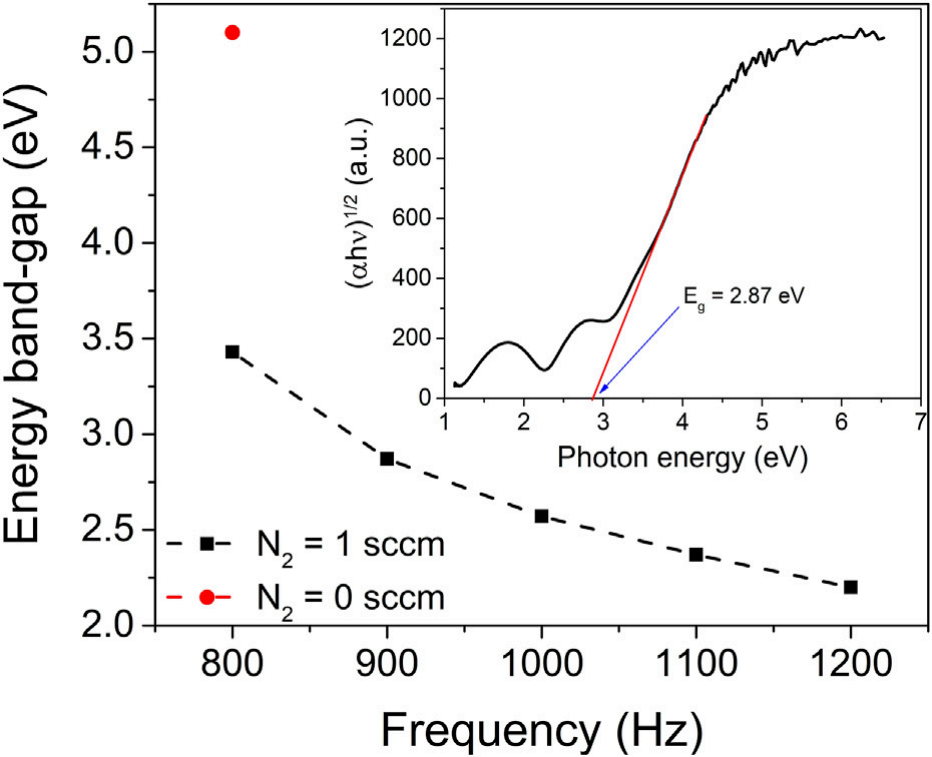
Optical band-gap of ZrO_2_ (red point) and ZrO_2-x_N_x_ thin films (black points) prepared at different pulsing repetition frequencies. The inset shows the Tauc plot used to estimate the optical band-gap of the ZrO_2-x_N_x_ thin film deposited at pulsing frequency of 900 Hz. The dashed line in the figure guides the eye to help visualize trends or patterns in the data.

To quantitatively determine the energy band-gap, the (αhν)^1/2^ vs. hν Tauc plot was utilized, with the linear portion of the plot being analysed. In the inset of Figure 5, this linear fit is presented, with the exponent of 1/2 chosen. This exponent is characteristic of the allowed indirect transition, which predominantly governs the optical absorption behaviour in zirconia thin films (Pinzón et al., 2014). By fitting the linear region, the band-gap energy of the ZrO_2_ and ZrO_2-x_N_x_ thin films can be accurately determined, providing insights into their optoelectronic properties and potential applications in various fields.

The obtained optical band-gap value of the zirconia thin film deposited solely in an Ar-O_2_ atmosphere (without nitrogen gas) aligns well with the literature-reported value for ZrO_2_ thin films (5.1 eV). This agreement indicates the reliability and accuracy of the deposition process and measurement techniques employed in this study.

As the pulsing repetition frequency increases from 800 to 1200 Hz, the energy band-gap of the ZrO_2-x_N_x_ thin films exhibits a gradual decrease from 3.43 to 2.20 eV. The narrowing of the band-gap with increasing repetition frequency (for *f* < 1000 Hz) can be attributed, with a high degree of certainty, to the variation in nitrogen content within the deposited films. The incorporation of nitrogen introduces deep-acceptor energy levels within the band-gap, in close proximity to the valence band (Kumari et al., 2015). This alteration in the energy levels within the band-gap leads to a narrowing of the optical band-gap. For repetition frequencies higher than 1000 Hz, the observed band gap narrowing, as the frequency increases, could be attributed to a phase change. This phase transition may be connected to the presence of Zr_2_ON_2_ and Zr_3_N_4_ phases, both of which have much lower band gap energies compared to ZrO_2_ or ZrO_2-x_N_x_. Previous reports have cited band gap values between 1.5 and 2.6 eV for Zr_2_ON_2_ (Thapa et al., 2022) and between 0.6 and 1.1 eV for Zr_3_N_4_ (Yablonskikh et al., 2014). Moreover, the optical properties of the thin films could also be influenced by the presence of internal stress. The combination of phase changes and internal stress within the films may collectively contribute to the observed alterations in their optical characteristics.

By narrowing the optical band-gap, the ZrO_2-x_N_x_ films exhibit improved light absorption in the visible region of the electromagnetic spectrum. This enhanced light absorption in the visible range is advantageous for photocatalytic applications, as it enables the utilization of a broader range of photons and facilitates more efficient utilization of solar energy. Consequently, the photocatalytic performance of ZrO_2-x_N_x_ thin films can be significantly improved, offering promising opportunities for their application as efficient photocatalysts in various environmental and energy-related processes.

#### 3.2.2 Chemical composition and bonding states

**FIGURE 6:**
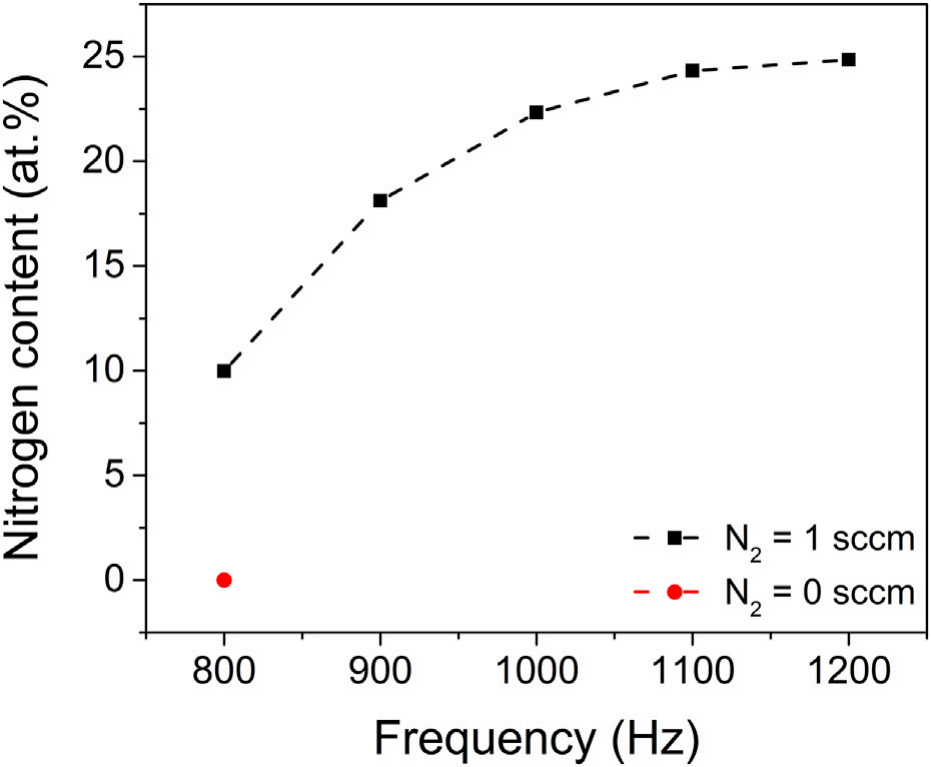
Nitrogen content in the ZrO_2_ (red point) and ZrO_2-x_N_x_ thin films (black points) prepared at different pulsing repetition frequencies. The dash-dotted line in the figure was drawn to guide the eye.

The nitrogen incorporation and chemical bonding states in the ZrO_2-x_N_x_ thin films were analysed using X-ray Photoelectron Spectroscopy (XPS). Figure 6 illustrates the nitrogen atom concentration within the ZrO_2-x_N_x_ films prepared at various pulsing repetition frequencies.

The XPS results reveal a clear trend: as the pulsing repetition frequency increases, the nitrogen content within the films also increases. This observation suggests that the nitrogen gas plays a crucial role in the incorporation of nitrogen atoms into the ZrO_2-x_N_x_ films during the deposition process.

Furthermore, it is noteworthy that the nitrogen content tends to approach a saturation value at high pulsing repetition frequencies, specifically above 1100 Hz. This implies that there might be an optimal range of pulsing repetition frequencies that allows for maximum nitrogen incorporation into the films. Beyond this range, further increasing the repetition frequency might not lead to significant changes in the nitrogen content.

The XPS analysis provides valuable insights into the nitrogen incorporation and chemical bonding states within the ZrO_2-x_N_x_ films, highlighting the influence of the pulsing repetition frequency on these properties. This information contributes to a better understanding of the film’s composition and can guide the optimization of deposition parameters for desired film characteristics.

Table 1 presents the estimated values for thickness, energy band-gap, and elemental composition of the ZrO_2-x_N_x_ thin films deposited at different pulse repetition frequencies.

**TABLE 1:** Measured values for thickness (*t*), energy band-gap (*E*_*g*_) and elemental composition (*at.%*) for the ZrO_2-x_N_x_ thin films deposited at different pulse repetition frequencies.

f (Hz)	800	900	1000	1100	1200
t (nm)	140	180	220	260	300
E_g_ (eV)	3.43	2.87	2.57	2.37	2.20
N (at.%)	10.0	18.1	22.3	24.3	24.9
O (at.%)	57.0	49.0	45.5	42.4	42.0
Zr (at.%)	33.0	32.9	32.2	33.3	33.1

At low pulsing repetition frequency values, the HiPIMS discharge operates in reactive mode, where the target surface is predominantly covered by metal oxide. This is because oxygen has a high reactivity, favouring the formation of metal-oxygen bonds over metal-nitrogen bonds. The combination of lower sputtering yield of metal oxide and the high reactivity of oxygen leads to less nitrogen incorporation into the growing film. In this regime, the presence of oxygen dominates the reaction, inhibiting the bonding between Zr and nitrogen atoms. As the pulsing repetition frequency increases, the target surface transitions from being predominantly covered by metal oxide to predominantly covered by metal nitride. This is attributed to the higher sputtering yield of metal nitride compared to metal oxide. Additionally, the limited amount of oxygen present in the working gas mixture (with a low O_2_ partial pressure of only 0.2%) contributes to an oxygen deficit in the deposited films. This oxygen deficit allows for the formation of bonding sites between the Zr atoms and the less reactive nitrogen atoms. The higher sputtering yield of the nitride is attributed to its characteristic binding energy, which is generally three times smaller than that of the corresponding oxide. However, as the pulsing repetition frequency is further increased, the nitrogen incorporation into the film tends to saturate. This is because the discharge transitions from a compound (nitride) sputtering mode to a metal sputtering mode, leading to a noticeable increase in the sputtered Zr atoms in the gas phase. At very low repetition frequencies (less than 200 Hz), a full oxidation of the growing film would be expected if the deposition rate is very low, and the available oxygen is sufficient to completely oxidize the sputtered Zr atoms.

It is important to note that the formation of metal nitrides is energetically less favourable than the formation of metal oxides due to the higher reactivity of oxygen. The enthalpy of formation of metal nitrides is generally lower than that of the corresponding oxide. Consequently, the formation of metal-oxygen bonds is energetically more favourable than metal-nitrogen bonds, and oxygen tends to prevail over nitrogen in the reaction. Overall, by controlling the pulsing repetition frequency in the HiPIMS process, it is possible to manipulate the surface composition and promote the incorporation of nitrogen into the deposited films, resulting in the formation of ZrO_2-x_N_x_ thin films with tailored properties for specific applications.

**FIGURE 7:**
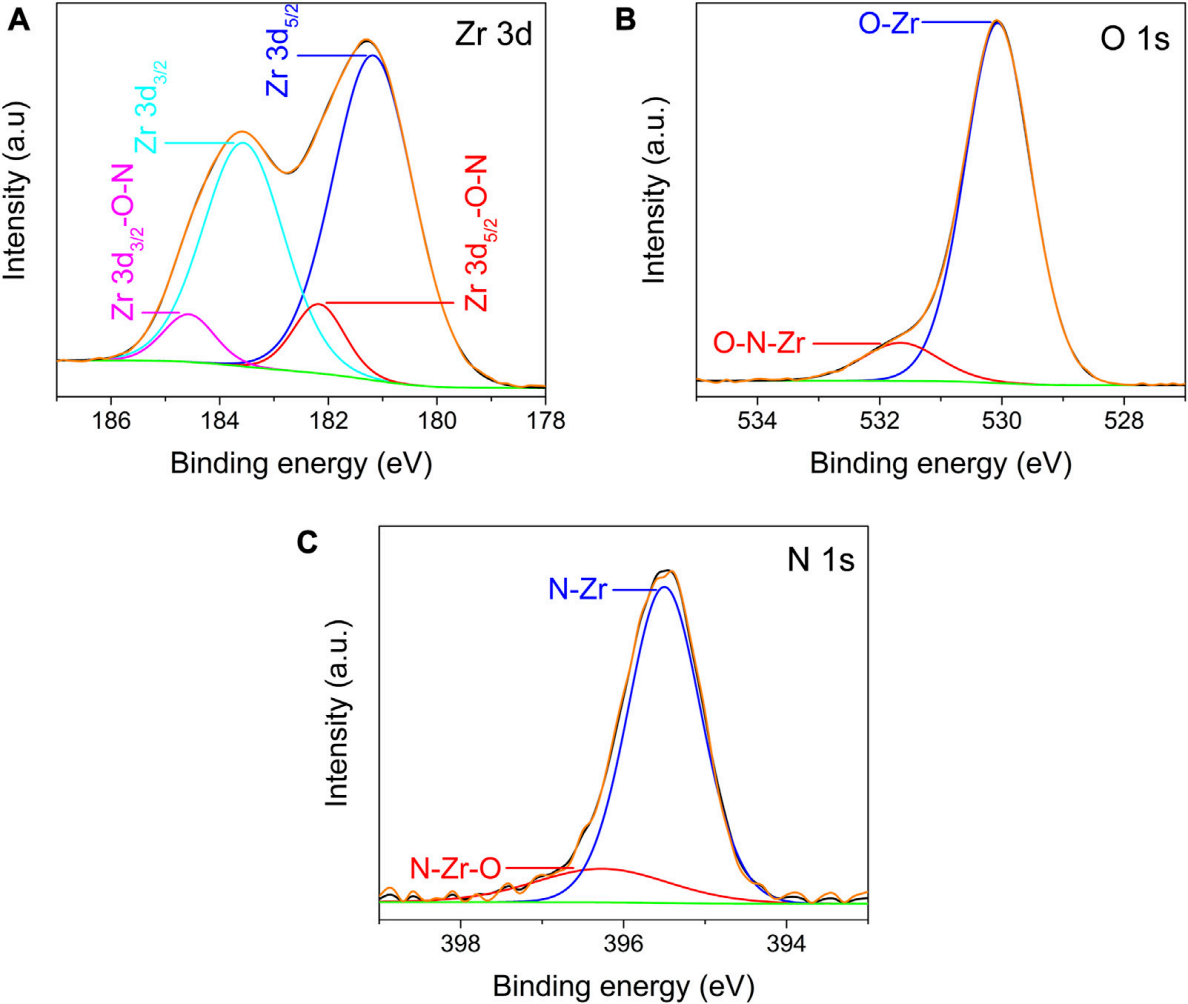
High resolution XPS spectra of Zr 3d **(A)**, O 1s **(B)** and N 1s **(C)** recorded from ZrO_2-x_N_x_ deposited at pulsing frequency of 900 Hz, after cleaning using Ar ion sputtering.

In the high-resolution XPS spectra of Zr 3d, O 1s, and N 1s recorded from the ZrO_2-x_N_x_ thin films deposited at a pulsing frequency of 900 Hz, after Ar sputter-etching, several important features were observed. Figure 7A shows the deconvolution of the Zr 3d spectrum, which resulted in four peaks corresponding to two pairs of doublets. The most intense doublet, with binding energies of approximately 181.2 eV and 183.6 eV, corresponds to the Zr 3d_5/2_ and Zr 3d_3/2_ core levels. These peaks are assigned to Zr^4+^ bonded to O^2-^, indicating the presence of ZrO_2_ in the thin film.

The lower doublet, positioned at around 182.2 eV and 184.6 eV, is attributed to Zr^4+^ bonded to O and N, suggesting the formation of Zr-O-N bonds in the ZrO_2-x_N_x_ lattice (Mejía et al., 2022). The presence of Zr-O-N bonds indicates that an oxygen atom is replaced by a nitrogen atom within the ZrO_2_ lattice. It is important to note that this lower doublet assigned to Zr-O-N bonds is absent in the high-resolution XPS spectra of Zr 3d recorded from the ZrO_2_ thin film, which was deposited without nitrogen.

The separation between the peaks of the doublets in the Zr 3d spectrum is approximately 2.4 eV. The less intense doublet, assigned to Zr-O-N bonds, has a slightly higher binding energy compared to the doublet associated with Zr-O bonds (ZrO_2_) due to the less electronegativity of nitrogen in Zr-O-N bonds. This observation is consistent with the formation of Zr-O-N bonds and further confirms the nitrogen incorporation into the ZrO_2-x_N_x_ thin films.

The high-resolution XPS spectra of O 1s and N 1s (Figures 7B,C) provide additional insights into the chemical states and bonding in the ZrO_2-x_N_x_ thin films.

In the high-resolution XPS spectrum of O 1s (Figure 7B), two distinct peaks were observed. The first peak, centred at around 530.1 eV, is attributed to O-Zr bonds within the ZrO_2_ lattice. This peak corresponds to the oxygen atoms bonded to zirconium in the ZrO_2_ structure. The second peak, detected at approximately 531.5 eV, has been assigned by various researchers to Zr-N-O bonds in the ZrO_2-x_N_x_ lattice (Cubillos et al., 2014a; Cubillos et al., 2017). This peak indicates the presence of oxygen atoms bonded to both zirconium and nitrogen atoms within the ZrO_2-x_N_x_ structure. The observation of the Zr-N-O bonding peak further confirms the nitrogen incorporation in the ZrO_2-x_N_x_ thin films.

Regarding the N 1s high-resolution XPS spectrum, it was deconvoluted considering two contributions (Figure 7C). The peak centred at around 395.5 eV is associated with N atoms four-coordinated to zirconium (N-Zr bonds) (Signore et al., 2007). This peak corresponds to nitrogen atoms directly bonded to zirconium atoms in the ZrO_2-x_N_x_ lattice. The peak detected at approximately 396.2 eV is tentatively assigned to Zr-N-O bonds in the ZrO_2-x_N_x_ lattice based on previous XPS data reported in the literature (Roman et al., 2011) and considering the XPS results discussed earlier. This peak represents the presence of nitrogen atoms bonded to zirconium and oxygen atoms within the ZrO_2-x_N_x_ structure. The broad width of this peak may be attributed to the presence of impurities on the film surface, such as oxy/carbo-nitrides. These impurities can influence the XRD peak characteristics and contribute to the observed broadening (Jaimes-Ochoa et al., 2019).

The deconvolution of the O 1s and N 1s high-resolution XPS spectra provides valuable information about the chemical bonding states in the ZrO_2-x_N_x_ films, confirming the presence of both Zr-N-O and N-Zr bonds in the lattice. These results, together with elemental composition listed in Table 1, support the successful incorporation of nitrogen into the ZrO_2_ lattice by oxygen substitution and the formation of ZrO_2-x_N_x_ films.

The narrowing of the bandgap by increasing the pulsing repetition frequency is strongly correlated with the variation of nitrogen content in the deposited films, resulting in the introduction of deep-acceptor energy levels in the bandgap (Kumari et al., 2015). This change in the energy bandgap can also be attributed to the covalent-bond character between nitrogen and oxygen, which reduces the strong electronegativity of oxygen in Zr-O ionic bonds observed in the ZrO_2_ lattice (Cubillos et al., 2014b). Additionally, the substitution of oxygen atoms with nitrogen atoms generates oxygen vacancies within the ZrO_2-x_N_x_ lattice, which, combined with the reduced bandgap, can significantly enhance the photocatalytic performance under visible light irradiation. This improvement is attributed to the increased absorption of visible light by the ZrO_2-x_N_x_ thin films, leading to enhanced photocatalytic activity.

#### 3.2.3 Structural analysis

**FIGURE 8:**
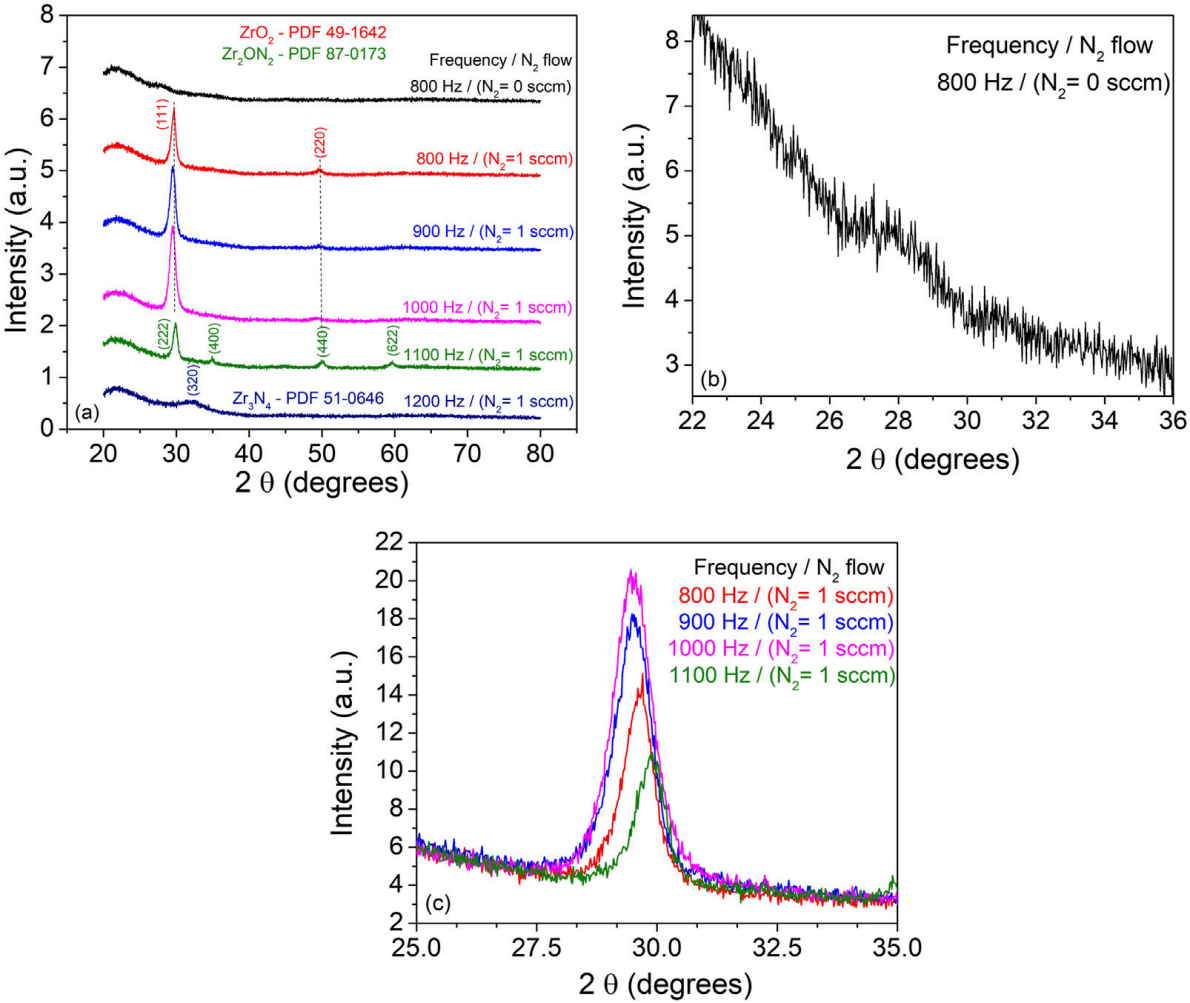
XRD patterns recorded from ZrO_2_ and ZrO_2-x_N_x_ thin films deposited on quartz substrates at room temperature and different pulse repetition frequencies.

The XRD analysis of the films deposited on quartz substrate revealed interesting findings, as shown in Figure 8. The diffractogram of the sample deposited without N_2_ predominantly exhibited an amorphous or near-amorphous microstructure, with a weak crystallographic signal at ∼27.8° that could correspond to the (111) main peak of the thermodynamically stable monoclinic ZrO_2_ structure. This suggests that the film deposited without nitrogen had limited crystallinity.

In contrast, for the film deposited in the presence of N_2_ at 800 Hz pulsing repetition frequency, two distinct diffraction lines were observed, indicating the presence of a distorted cubic ZrO_2_ phase. The shift of the XRD peaks towards lower 2θ angles compared to the polycrystalline bulk position can be attributed to the energetic ion bombardment during the film deposition process (Hovsepian et al., 2014; Cemin et al., 2019). The inclusion of nitrogen in the zirconium oxide structure can also contribute to compressive residual stress within the film, further shifting the diffraction lines (Nordseth et al., 2019). With increasing pulsing repetition frequencies up to 1000 Hz, the intensity and displacement of the diffraction from the (111) crystallographic plane became more pronounced. This suggests a preferential crystallographic ordering and a possible improvement in crystallinity. However, it should be noted that the increasing thickness of the samples could also contribute to the higher peak intensity. For the sample deposited at 1100 Hz, the observed diffraction lines were assigned to the cubic Zr_2_ON_2_ phase and the wide peak at ∼32.14° was associated with the (320) crystallographic plane of the Zr_3_N_4_ structure. These results are consistent with the XPS measurements, which indicated an increase in nitrogen content with the pulsing repetition frequency.

The estimated crystallite average size, ranging between 8 and 12 nm, using Scherrer’s equation and the Pseudo-Voigt fit of the main peaks, showed no dependency on the pulsing repetition frequency. This suggests that the crystallite size was not significantly affected by the variations in the deposition conditions.

Overall, the XRD analysis provided valuable insights into the structural properties and phase composition of the deposited films, confirming the presence of different phases and crystallographic structures depending on the deposition conditions and nitrogen incorporation.

The results illustrated and discussed in the previous sections concerning the influence of the repetition frequency on the measured deposition rate, intensity of Zr^0^ optical emission spectral line and target peak current allow a more comprehensive understanding of the variation in the stoichiometry of the films.

#### 3.2.4 Photocatalytic activity assessment

**FIGURE 9:**
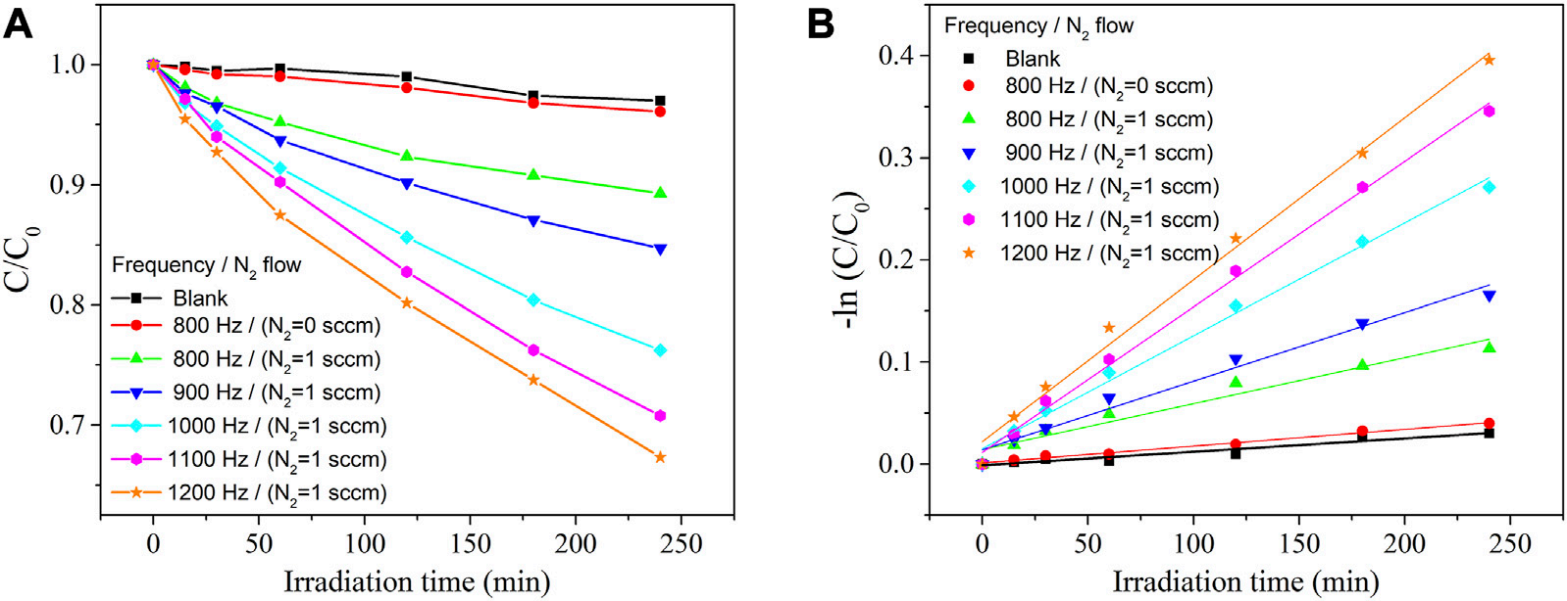
Degradation of MB dye under visible-light irradiation in the absence (blank) and presence of catalysts **(A)** and its pseudo-first-order kinetic analysis **(B)**.

The photocatalytic activity of the ZrO_2-x_N_x_ films was evaluated by studying the photodegradation of the organic MB dye under visible-light irradiation. Figure 9A presents the change in MB concentration as a function of irradiation time for the ZrO_2-x_N_x_ catalysts obtained under different plasma processing conditions. In the absence of a photocatalyst (blank experiment), no significant changes in the MB concentration were observed under visible-light irradiation, indicating that the dye does not undergo spontaneous degradation in the absence of a catalyst.

The ZrO_2_ thin film, with its wide optical band-gap (E_g_ = 5.1 eV), exhibited a very low decomposition rate, where only approximately 4% of the initial organic dye was decomposed after 4 h of visible-light irradiation (λ > 400 nm). This confirms the limited photocatalytic activity of ZrO_2_ under visible-light conditions due to its large band-gap.

In contrast, improved photocatalytic activity was observed for the N-doped zirconia samples, which can be attributed to the narrowing of the band-gap. As mentioned earlier, increasing the pulsing repetition frequency led to an increase in the nitrogen content in the films and a gradual decrease in the optical band-gap. This band-gap narrowing is believed to enhance the absorption of visible light and promote the generation of photoexcited charge carriers, thus facilitating the photocatalytic degradation of MB.

The results of the photocatalytic tests demonstrate the potential applicability of the ZrO_2-x_N_x_ films as visible-light-driven photocatalysts for organic dye degradation. The band-gap engineering through nitrogen incorporation offers a promising strategy for enhancing the photocatalytic performance of ZrO_2_-based materials.

The narrowing of the band-gap in ZrO_2-x_N_x_ films through nitrogen doping enables the absorption of a greater number of visible-light photons, leading to the generation of more electron-hole pairs and improved photocatalytic behaviour. As shown in Figure 9, the ZrO_2-x_N_x_ films with narrower band-gaps (films deposited at higher pulsing frequencies) exhibit higher efficiency as photocatalysts for the degradation of MB dye under visible-light irradiation.

**FIGURE 10:**
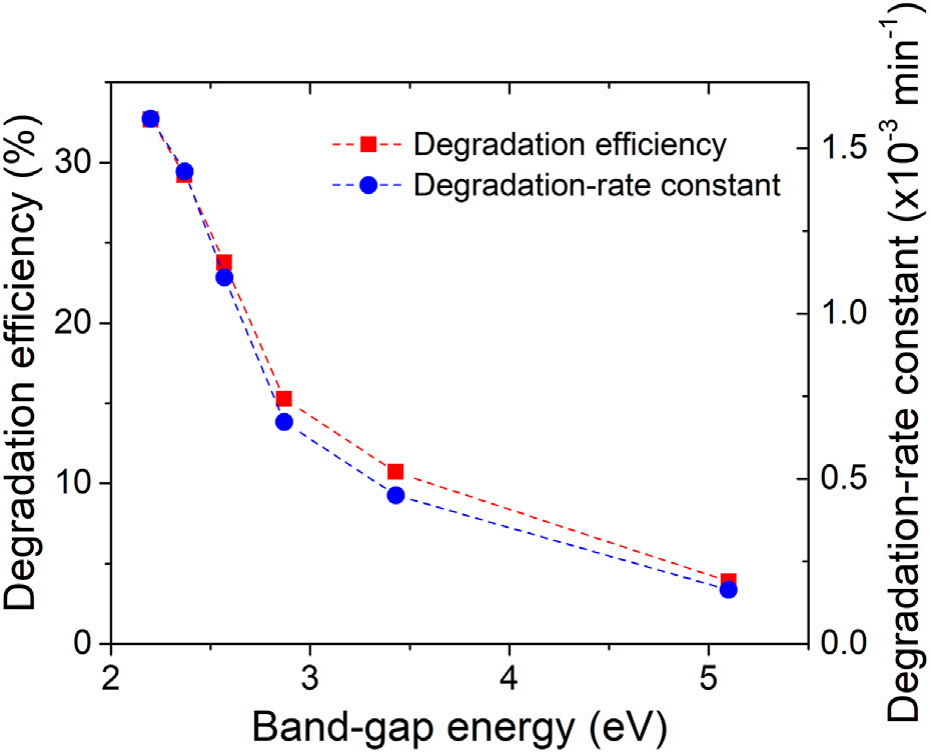
Degradation efficiency and degradation-rate constant vs. energy band-gap of ZrO_x_N_y_ thin films.

To quantitatively analyse the degradation kinetics, the MB degradation-rate constant (k) was determined by fitting the kinetic data obtained from the linear fitting curves of -ln(C/C0) versus irradiation time (Figure 9B). The excellent linearity of the plots indicates that the photodegradation rate of MB follows a pseudo-first-order kinetic reaction: -ln(C/C0) = kt, where k represents the photodegradation-rate constant (Nazir et al., 2021). Furthermore, the calculated MB degradation efficiency and degradation-rate constant (k) were plotted against the energy band-gap of the ZrO_2-x_N_x_ samples in Figure 10. It can be observed that as the energy band-gap decreases (due to nitrogen doping), the MB degradation efficiency and the degradation-rate constant increase. This correlation confirms the influence of the band-gap on the photocatalytic performance of ZrO_2-x_N_x_ films. The narrower band-gap allows for more efficient utilization of visible light and promotes higher photocatalytic activity.

These findings highlight the potential of nitrogen-doped ZrO_2-x_N_x_ films as effective photocatalysts for visible-light-driven degradation of organic dyes, offering a promising avenue for various environmental and energy-related applications.

The decrease in the energy band gap of ZrO_2-x_N_x_ films from 5.1 to 2.2 eV leads to a gradual increase in MB degradation efficiency from 4.0% to 32.7% after 4 h of visible-light irradiation. Simultaneously, the degradation-rate constant increases by a factor of ten. It is important to note that the efficiency of the films is lower compared to zirconia nanoparticles (Teeparthi et al., 2018) due to their lower specific surface area, which limits the number of available active sites for photocatalytic reactions.

Figure 10 further illustrates the relationship between enhanced photocatalytic activity and the decreasing band gap in N-doped zirconia. The narrower band gap allows for better utilization of visible light, facilitating the migration of more photo-excited electrons from the valence band (VB) to the conduction band (CB) of the catalyst. This enhanced migration leads to the generation of more holes in the VB, which can react with adsorbed water molecules on the catalyst’s surface to produce hydroxyl radicals (^●^OH). Additionally, the conduction band electrons can react with dissolved oxygen to generate superoxide anions (O_2_^−^). The combined presence of VB holes, hydroxyl radicals, and superoxide anions acts as oxidizing and reducing agents in the decomposition reactions of organic contaminants.

These mechanisms highlight the role of nitrogen doping in promoting visible-light-driven photocatalytic activity by enabling efficient utilization of light energy and facilitating the generation of reactive species for organic degradation. While the films may have lower efficiency compared to nanoparticles, their unique properties and potential for integration into various applications make them promising materials for future photocatalysis studies.

Indeed, while the optical band gap energy of the ZrO_2-x_N_x_ photocatalyst plays a significant role in determining the degradation efficiency, it is important to consider that other factors can also influence the overall photocatalytic performance. Morphology, size, and crystallinity of the photocatalyst are additional factors that can impact photocatalytic activity (Tiron et al., 2022). These factors affect the specific surface area, surface reactivity, charge carrier mobility, and light absorption properties of the material, all of which contribute to the overall photocatalytic performance.

In conclusion, the narrow band gap of the ZrO_2-x_N_x_ thin film obtained through reactive HiPIMS makes it a promising candidate for visible-light-driven photocatalytic decomposition of organic contaminants. However, to fully optimize the photocatalytic performance, it is crucial to consider and optimize other factors such as morphology, size, and crystallinity, along with the band gap energy. Further research and development in these areas will contribute to the advancement of efficient and effective photocatalytic materials for environmental remediation applications.

## 4 Conclusion

The incorporation of nitrogen into ZrO_2_ thin films using reactive HiPIMS with carefully selected process conditions allows for precise control over the nitrogen content. By adjusting the pulsing repetition frequency, a gradual transition from compound to metal sputtering mode is achieved, effectively suppressing hysteresis, and ensuring process stability. The reactive gas mixture, consisting of Ar, N_2_, and O_2_, combined with short pulse durations, facilitates this transition.

The introduction of nitrogen into the crystal structure of the thin films leads to significant changes in their optical properties. The decrease in band-gap energy is directly correlated to the nitrogen content in the films. By varying the pulsing frequency within the range of 800–1200 Hz, crystalline ZrO_2-x_N_x_ thin films with nitrogen contents ranging from approximately 10–25 at.% and optical band gaps ranging from 3.43 to 2.20 eV can be deposited at room temperature.

The suitability of ZrO_2-x_N_x_ thin films for visible-light driven photocatalysis was verified by conducting photodegradation tests with methylene blue (MB) organic dye as a representative contaminant. The results revealed that the photocatalytic activity of N-doped zirconia improves with an increase in N content. This enhancement in photocatalytic performance is attributed to the narrower energy band gap achieved through nitrogen doping. The ability to tune the nitrogen content in the films provides a means to optimize the photocatalytic performance.

The chemical composition and deposition rate of the coatings can be analytically described based on the variation of the peak current, as supported by experimental findings. This analytical approach offers insights into the relationship between deposition parameters and film properties.

The combination of a narrow band-gap energy, increasing deposition rate, and stable deposition process makes this research a promising solution for addressing energy and environmental challenges. It sheds light on new trends and strategies for developing innovative technologies in the synthesis and doping of advanced photocatalytic materials.

## Data Availability

The original contributions presented in the study are included in the article/Supplementary Material, further inquiries can be directed to the corresponding author.
