# Supplementary material for: Band-gap engineering of zirconia by nitrogen doping in reactive HiPIMS: a step forward in developing innovative technologies for photocatalysts synthesis
Source: Front Chem. 2023 Aug 10;11:1239964. doi: 10.3389/fchem.2023.1239964 (PMC10448390; doi:10.3389/fchem.2023.1239964)
Supplement: Supplementary file 1 [file DataSheet1.pdf]

Supplementary Material

Band-gap engineering of zirconia by nitrogen doping in reactive HiPIMS: A step forward in developing innovative technologies for photocatalysts synthesis

Teodora Matei[1], Vasile Tiron[2], Roxana Jijie[2], Georgiana Bulai[3], Ioana-Laura Velicu[1], Daniel Cristea[4], Valentin Crăciun[5,6]

*** Correspondence:** Ioana-Laura Velicu: laura.velicu@uaic.ro

1 Supplementary Figures and Tables

1.1 Supplementary Figures

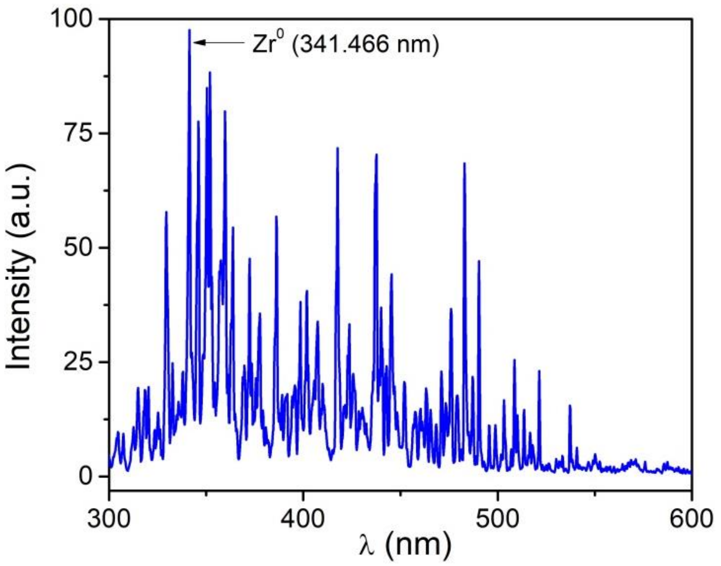

Figure S1. Optical emission spectrum in the range 300–600 nm of plasma during HiPIMS of Zr target in argon, nitrogen, and oxygen gas mixture at pressure 0.8 Pa and repetition frequency 1 kHz.

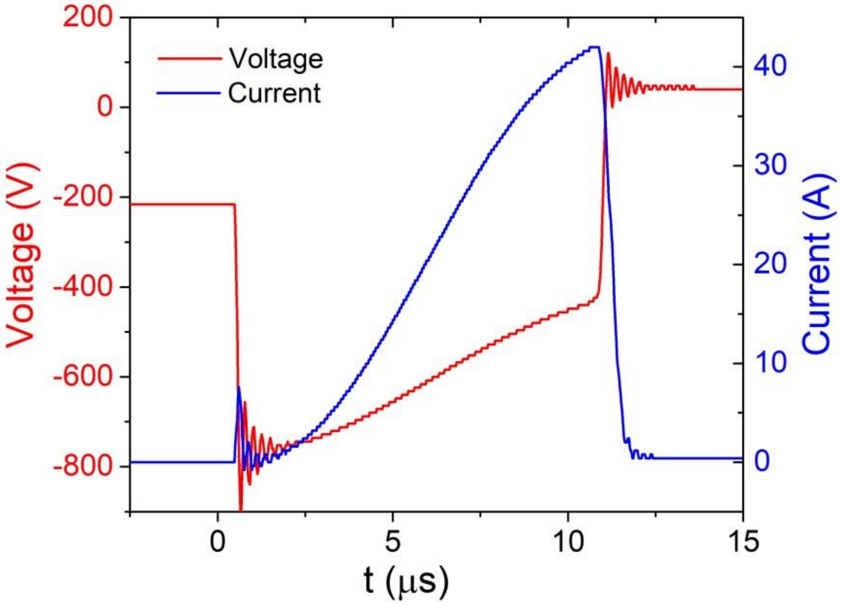

Figure S2. Time evolution of the target voltage and discharge current during HiPIMS of Zr target in argon, nitrogen, and oxygen gas mixture at pressure 0.8 Pa and repetition frequency 1 kHz.
